# Stress-related growth among university athletes following COVID-19-related stress: between-wave differences in associations with meaning-making and perceived available support in sport

**DOI:** 10.3389/fpsyg.2026.1778982

**Published:** 2026-10-20

**Authors:** Kojiro Matsuda, Yasuo Susaki, Yoshio Sugiyama, Ethan Sahker, Eriko Aiba

**Affiliations:** 1 Faculty of Management, Josai University, Sakado, Japan; 2 Faculty of Education, Shimane University, Matsue, Japan; 3 Faculty of Human-Environment Studies, Kyushu University, Kyushu, Japan; 4 Population Health and Policy Research Unit, Center for Medical Education and Internationalization, Graduate School of Medicine, Kyoto University, Kyoto, Japan; 5 College of Education, Yokohama National University, Yokohama, Japan

**Keywords:** benefit finding, meaning reconstruction, multigroup simultaneous analysis, repeated cross-sectional study, resilience

## Abstract

**Introduction:**

In the present study, stress-related growth (SRG) was operationalized as athletes’ self-perceived growth following a stressful experience in sport. This study examined whether the associations of meaning-making processes (assimilation and accommodation) and perceived available support in sport (PASS) with SRG differed between two independent samples of university athletes who reported COVID-19-related stressful experiences in sport.

**Methods:**

Meaning-making was assessed retrospectively with reference to participants’ thoughts at the beginning of a COVID-19-related stressful experience; SRG assessed perceived change since before that experience; and PASS assessed satisfaction with sport-related support during these COVID-19 contexts. Using a repeated cross-sectional design, we compared two independent samples: T1, collected in September–October 2023 (*N* = 145), and T2, collected in July 2025 (*N* = 150). Multigroup structural equation modeling was used to examine between-wave differences in the structural associations among PASS, assimilation, accommodation, and SRG.

**Results:**

At T1, accommodation was significantly associated with SRG, whereas assimilation was not; at T2, assimilation was significantly associated with SRG, whereas accommodation was not. PASS showed a large and stable direct association with SRG at both time points. Formal between-wave tests identified significant differences for the PASS-to-accommodation and accommodation-to-SRG paths, but not for the assimilation-to-SRG path. Overall, the two independent samples showed different association patterns between meaning-making and SRG.

**Discussion:**

The findings suggest that meaning-making processes may be related to SRG differently across the two independent samples, whereas sport-related support showed, a consistent positive association with SRG. For university athletics, these results highlight the potential relevance of maintaining supportive interpersonal environments when athletes are adapting to prolonged stress and adversity.

## 1 Introduction

### 1.1 Student-athlete adversity and the COVID-19 context

The COVID-19 pandemic disrupted sport participation and social contact for many student-athletes, although the form and intensity of these effects varied across countries, institutions, sports, and phases of the crisis. For example, campus closures and physical-distancing measures separated collegiate athletes from teammates and interrupted sport participation, with implications for athletic identity and mental health (Graupensperger et al., 2020). Other studies have documented mental health difficulties among athletes during and after the pandemic (Batalla-Gavalda et al., 2021; Martínez-González et al., 2021; Collins et al., 2022; Bird et al., 2024; Strauser et al., 2025). Against this background, the present study focuses on Japanese university athletes who reported COVID-19-related stressful experiences in sport.

### 1.2 Stress-related growth (SRG)

Stress-related growth (SRG) broadly refers to positive changes associated with stressful experiences, as reflected in Park et al.’s (1996) Stress-Related Growth Scale, which was developed to assess stress-related positive outcomes in college students. In sport research, Kemuriyama and Amazaki (2015) developed the Stress-Related Growth Scale for Athletes (SRGSA) and conceptualized SRG in the sport context as a sense of growth following stressful experiences in sport. Accordingly, the present study operationalized SRG as athletes’ perceived growth following COVID-19-related stressful experiences in sport, as assessed by the SRGSA. SRG and posttraumatic growth (PTG) are related but are not treated as interchangeable in this study. Tedeschi and Calhoun (2004), p. 1 defined posttraumatic growth as “the experience of positive change that occurs as a result of the struggle with highly challenging life crises,” and their Posttraumatic Growth Inventory assesses five domains: new possibilities, relating to others, personal strength, spiritual change, and appreciation of life (Tedeschi and Calhoun, 1996). We focus on SRG because participants reported COVID-19-related stressful experiences in sport that varied in severity and were not assumed to constitute trauma.

Positive change after adversity has been reported in sport across various contexts, including following injury (Wadey et al., 2011; Salim et al., 2016; Roy-Davis et al., 2017), the yips (Matsuda et al., 2018), deselection (Neely et al., 2018), interpersonal conflict and abuse (Tamminen et al., 2013), and major performance failure (Howells and Fletcher, 2015; Sarkar et al., 2015). Howard-Cook and Howells (2024) extended this literature to the COVID-19 context. Five elite athletes completed the Stress-Related Growth Scale–Revised and semistructured interviews, and thematic analysis identified both illusory and real adversarial growth, with well-being integral across their accounts. Their findings support examining athletes’ reports of positive change after COVID-19 while remaining cautious about what self-reported growth represents.

### 1.3 Meaning-making

Park’s (2010) integrated model distinguishes global meaning from situational meaning and distinguishes meaning-making efforts from meaning made. When the appraised meaning of an event conflicts with broader beliefs or goals, that discrepancy can motivate meaning-making efforts (Park and Folkman, 1997; Park, 2010). The present study operationalizes meaning-making with Horita and Sugie’s (2013) Assimilation and Accommodation of Meaning Making Scale (AAMS). Horita and Sugie (2013), p. 111 conceptualized assimilation as naturally interpreting and understanding an experience in a manner consistent with one’s existing ways of seeing and thinking, whereas accommodation involves consciously and intentionally engaging in cognitive and emotional processing to understand and interpret the experience while revising those existing perspectives. Horita and Sugie (2013), citing Block (1982), further note that assimilation and accommodation are not mutually exclusive and may occur simultaneously. Accordingly, the present study does not assume that they follow a fixed developmental sequence.

### 1.4 Sport-related social support (PASS)

Social support provides interpersonal resources that can be relevant when people manage stress (Cohen and Wills, 1985; Thoits, 2011). In sport psychology, perceived available support has been conceptualized as a recipient’s judgment that relevant others would provide assistance if needed, and the PASS-Q assesses emotional, esteem, informational, and tangible support (Rees and Hardy, 2000; Freeman et al., 2011). In the present study, perceived available support in sport (PASS) was operationalized with the Athletic Social Support Scale (ASSS; Tsuchiya and Nakagomi, 1994), administered as ratings of satisfaction with sport-related support; the specific subscales and instructions are described in Section 2.4.3. Sport research also links social support with positive psychological change following adversity. In qualitative work with Division I intercollegiate athletes, social support emerged as one of four dimensions of the SRG process and was described as critical in helping athletes work through and make meaning from their stressors (Galli and Reel, 2012); in a prospective study of athletic injury, appraisal support significantly predicted subsequent PTG (Nakamura and Tsuchiya, 2020).

Both social support and meaning-making have been examined in relation to adjustment following stressful experiences (Park, 2010). Horita (2016) also examined social support as a situational factor in relation to assimilation and accommodation. In the present model, PASS, assimilation, accommodation, and SRG are therefore examined as associated constructs. Because these variables were measured within each cross-sectional wave, any indirect effects are interpreted as model-implied indirect associations rather than evidence that PASS causes SRG through meaning-making.

### 1.5 Research gap

Research on athletes during and after COVID-19 has predominantly examined distress, including anxiety, depression, negative mood, and disruption of athletic identity (Graupensperger et al., 2020; McGuine et al., 2021; Mehrsafar et al., 2021; Gouttebarge et al., 2022; Bird et al., 2024). Positive change has received less attention. Howard-Cook and Howells (2024) provided qualitative evidence of growth-related experiences among elite athletes following the pandemic, but quantitative evidence on SRG among university athletes remains limited. In addition, few studies have integrated sport-specific social support with the distinct meaning-making processes of assimilation and accommodation in relation to SRG.

The present data were collected at two survey time points, Time 1 (T1; September–October 2023) and Time 2 (T2; July 2025), using independent samples. These time points should not be interpreted as successive developmental stages. Moreover, at both waves, the meaning-making measure asked participants to recall their thoughts at the beginning of their COVID-19-related stressful experience rather than to report current meaning-making. Consequently, T1–T2 differences may reflect retrospective recall or reconstruction, cohort or sample composition, institutional or sporting context, or other between-sample factors.

### 1.6 The present study

Using a repeated cross-sectional design and multigroup structural equation modeling, this study examined whether the structural associations of assimilation, accommodation, and PASS with SRG differed between two independent samples of Japanese university athletes who reported COVID-19-related stressful experiences in sport. The study was designed to compare between-wave association patterns; it was not designed to estimate within-person development or causal change over time.

Hypothesis 1 (Between-Wave Differences in Meaning-Making Associations). The accommodation–SRG association was expected to be stronger at T1 than at T2, whereas the assimilation–SRG association was expected to be stronger at T2 than at T1. This is a group-level hypothesis concerning differences between independent samples, not a claim that individual athletes move sequentially from accommodation to assimilation.

Hypothesis 2 (Direct and Indirect Associations of PASS). PASS was expected to show a positive direct association with SRG at both time points, together with positive model-implied indirect associations with SRG through assimilation, accommodation, or both. Because the design is cross-sectional within each wave, these indirect associations are not interpreted as evidence of temporal or causal mediation.

The integrated hypothetical model is shown in Figure 1.

**Figure 1:**
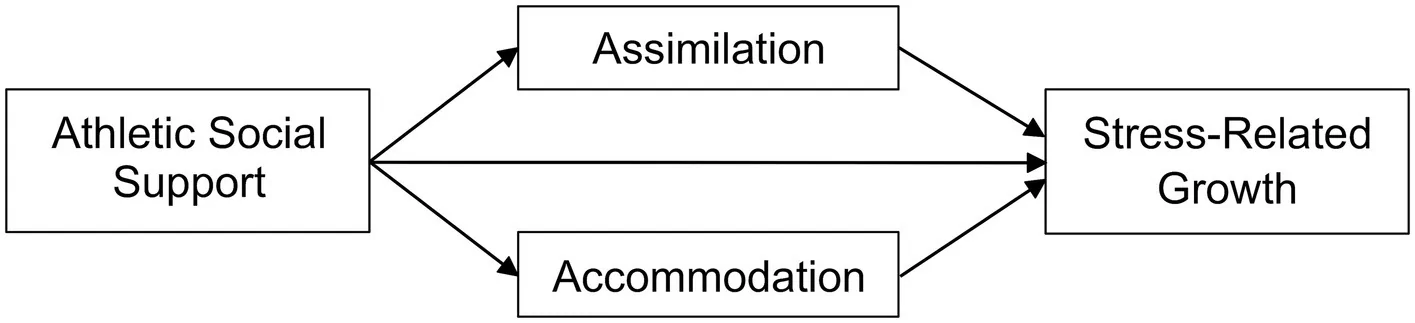
Hypothetical model of the study.

## 2 Materials and methods

### 2.1 Research design

This study employed a repeated cross-sectional design (Menard, 2008), comparing independent samples assessed at T1 (September–October 2023) and T2 (July 2025); participants were not linked across waves. This design permits comparisons of association patterns between samples but cannot estimate within-person change, establish temporal precedence among variables, or support causal inference. This limitation is particularly important because meaning-making was assessed retrospectively at both waves with reference to the beginning of participants’ COVID-19-related stressful experiences. Accordingly, between-wave differences may reflect retrospective recall or reconstruction, cohort or contextual differences, institutional or sport-composition differences, rather than psychological change within the same athletes over time.

To characterize the comparability of the two independent samples, we compared demographic and sport characteristics across waves. At each time point, anonymous, individually completed questionnaires were administered in person at participating universities, primarily during university lectures or after practice sessions, under the direct supervision of the first author or trained research collaborators. Google Forms was used solely as the response platform during these supervised sessions. Responses were collected after oral and written explanations of the research purpose, confidentiality, the voluntary nature of participation, the right to withdraw, and the fact that participation or withdrawal would have no impact on academic or athletic activities.

### 2.2 Participants and implementation procedures

The inclusion criteria for participants in the analysis were as follows: (1) being a current university student at the times of the survey (T1: September–October 2023; T2: July 2025); (2) having continuously participated in organized sport activities from March 11, 2020, until the time of the survey; (3) being affiliated with a sports team or club and engaging in competitive activities, or regularly practicing a sport as an individual competitor (excluding recreational participation); and (4) having responded “Yes” to the question, “Have you experienced any stressful events related to COVID-19 in your club activities or sports activities?” Importantly, criterion (2) refers to the continuity of overall sport participation rather than the continuity of university enrollment. Participants were excluded primarily because they did not meet one or more of the following criteria: continuous organized sport participation since March 2020, competitive or structured sport involvement, or self-reported COVID-19-related stressful experience in sport. Because recruitment was conducted through open invitations in university sport contexts, the total number of eligible athletes invited could not be determined; therefore, formal response rates and comparisons between included and excluded respondents were unavailable. This should be considered when interpreting potential selection bias. Because most participants had already been continuously engaged in organized sports prior to entering university (e.g., during high school), this criterion did not imply that T2 participants had been enrolled at the university for more than 5 years. Note that criterion (4) is intended to confirm the presence or absence of stress experiences related to COVID-19 through self-report. These criteria targeted university athletes with continuous sports participation since the declaration of the pandemic, rather than continuous university enrollment. Comparisons of age, sex, grade level, and sport type between the two analytic samples are summarized in Table 1.

**Table 1:** Demographic comparability between T1 and T2 samples.

Variable	Test	Statistic	df	*p*	Effect size	Interpretation
Age (years)	*t* (Welch)	*t* = 1.723	291.3	0.086	d = 0.20 (small)	No significant difference
Sex	χ^2^(1)	χ^2^ = 2.125	1	0.145	V = 0.06 (very small)	No significant difference
Grade level	χ^2^(3)	χ^2^ = 7.387	3	0.061	V = 0.12 (small)	No significant difference
Sport type	χ^2^(26)†	χ^2^ = 113.789	26	<0.001	V = 0.55 (large)	Substantial difference

^†^Fisher’s exact test (Monte Carlo, B = 10,000): *p* = 0.0001. Age: T1 M = 19.59 (SD = 1.06), T2 M = 19.36 (SD = 1.19). Sex: T1 *n* = 95 male/50 female; T2 *n* = 110 male/40 female. Sport type non-equivalence is driven by the overrepresentation of soccer players at T2 (*n* = 86, 57.3%) relative to T1 (*n* = 21, 14.5%). This composition difference is directly addressed by the sensitivity analyses reported in §3.9.

### 2.3 Ethical statement

This study received approval from the ethics committee of Kumamoto Gakuen University. All participants were university students aged 18–22 years and were adults at the time of participation. Before participating, all participants received oral and written information about the study’s purpose, potential risks, confidentiality, voluntary participation, and data use. Written informed consent was obtained directly from each participant; consent from legal guardians or next of kin was not required. Only those who understood and agreed to the research outline and ethical considerations completed the questionnaire.

### 2.4 Measurement scales

Participants provided basic sociodemographic information, including age, biological sex, and grade level, as well as details about their sports experience, such as sport discipline and competitive history. Additionally, they shared their experiences related to COVID-19 and completed three established psychological scales. All three psychological measures were administered in Japanese; English translations of the sample items and instructions are provided below for reporting purposes.

#### 2.4.1 SRG

The Stress-Related Growth Scale for Athletes (SRGSA; Kemuriyama and Amazaki, 2015) was developed to assess SRG in sport. In developing the scale, Kemuriyama and Amazaki (2015), p. 146 conceptualized SRG in the sport context as a sense of growth following a stressful experience in sport. This 15-item scale consists of three factors: understanding and empathy toward teammates (five items), stress management skills (five items), and attitude change toward sport (five items).

Sample questions for understanding and empathy include, “I have become more respectful of my relationships with my teammates,” and “I have become more aware of my teammates’ problems as if they were my own.” For stress management skills, sample questions include, “I have become more proactive in my efforts and see stress problems as a process of growth,” and “I have become more patient and persistent in my efforts even when things don’t go well.” Regarding attitude change toward sports, sample questions include, “I am now able to prepare my mind and body for competition,” and “I have started to organize my problems in sport and set new goals.”

Responses to these items are scored on a 5-point Likert scale, ranging from 1 (not at all applicable) to 5 (very applicable). In the present study, separate scores were calculated for the three subscales: understanding and empathy toward teammates, stress management skills, and attitude change toward sport. Kemuriyama and Amazaki (2015) confirmed the scale’s reliability and validity. In the present samples, Cronbach’s α values for understanding and empathy toward teammates, stress management skills, and attitude change toward sports were 0.91, 0.88, and 0.81 at T1, and 0.89, 0.87, and 0.83 at T2, respectively (Table 2).

**Table 2:** Descriptive statistics, reliability coefficients, and intercorrelations among study variables at T1 and T2.

Variables	T1 (*n* = 145)			T2 (*n* = 150)			*t*	1	2	3	4	5	6	7	8	9
	M	SD	α	M	SD	α										
Athletic social support																
1. Affective support	37.05	6.60	0.95	36.83	5.58	0.90	0.31	—	0.83**	0.82**	0.72**	0.40**	0.42**	0.49**	0.54**	0.53**
2. Companionship	24.87	4.19	0.89	24.40	3.75	0.81	1.01	0.88**	—	0.69**	0.68**	0.40**	0.40**	0.48**	0.58**	0.44**
3. Support for self-esteem	16.73	3.00	0.91	16.49	2.85	0.82	0.72	0.89**	0.83**	—	0.70**	0.36**	0.37**	0.46**	0.45**	0.49**
4. Instructive support	20.30	3.36	0.85	20.11	3.09	0.79	0.49	0.90**	0.83**	0.83**	—	0.40**	0.38**	0.48**	0.57**	0.49**
Meaning-making																
5. Assimilation	25.41	4.77	0.87	23.99	5.73	0.84	2.32*	0.39**	0.33**	0.35**	0.37**	—	0.78**	0.53**	0.54**	0.46**
6. Accommodation	26.94	4.43	0.91	26.05	5.19	0.80	1.57	0.32**	0.28**	0.29**	0.36**	0.73**	—	0.51**	0.46**	0.41**
Stress-related growth for athletes																
7. Understanding and empathy toward teammates	21.19	3.99	0.91	20.91	4.13	0.89	0.61	0.56**	0.54**	0.53**	0.60**	0.46**	0.57**	—	0.63**	0.57**
8. Stress management skills	20.31	3.87	0.88	19.61	4.26	0.87	1.48	0.55**	0.48**	0.49**	0.62**	0.54**	0.58**	0.50**	—	0.59**
9. Attitude change Toward Sports	20.10	3.89	0.81	19.58	4.18	0.83	1.10	0.54**	0.48**	0.46**	0.60**	0.48**	0.44**	0.53**	0.71**	—

M = mean; SD = standard deviation; α = Cronbach’s alpha; *t* = *t* statistic for the T1–T2 mean comparison. Correlation coefficients are presented with the T2 values in the upper entry and the T1 values in the lower entry. T1 data were collected in September–October 2023; T2 data were collected in July 2025. **p < 0.01, *p < 0.05.

Participants completed the SRGSA after reading the following instructional text: “After experiencing a stressful event related to COVID-19 in a sports context, how have you changed since before the event?” They selected one response for each item on a 5-point Likert scale ranging from 1 (not at all applicable) to 5 (very applicable). Thus, SRG was assessed as participants’ current, self-perceived change relative to a recalled pre-event baseline, rather than as a contemporaneous measure obtained at the time of the stressful experience.

#### 2.4.2 Meaning-making

Following Horita and Sugie (2013), the 14-item Assimilation and Accommodation of Meaning Making Scale (AAMS) was used to assess two meaning-making processes, each represented by seven items. Following the original definitions reported by Horita and Sugie (2013), p. 111, assimilation was operationalized as naturally interpreting and understanding an experience in a manner consistent with one’s existing ways of seeing and thinking, whereas accommodation was operationalized as consciously and intentionally engaging in cognitive and emotional processing to understand and interpret the experience while revising those existing perspectives. Assimilation is evaluated with statements such as, “The meaning of the experience is consistent with my way of seeing and thinking about things,” and “I can use my perspectives and ideas to help me understand that experience.” In contrast, accommodation is assessed with items such as, “I feel the need to reassess my own way of seeing and thinking about things,” and “I am trying to make sense of this experience in some way.” Participants rate each item on a 5-point Likert scale, ranging from 1 (strongly disagree) to 5 (strongly agree). The subscale scores were calculated. Horita and Sugie (2013) established the scale’s construct validity and reliability. In the present samples, Cronbach’s α values for assimilation and accommodation were 0.87 and 0.91 at T1, and 0.84 and 0.80 at T2, respectively (Table 2).

Participants completed the scale after reading the following instruction: “How much did you think about the following at the beginning of your experience of a stressful event related to COVID-19 in your sport activity? Reflecting on your thoughts at that time, please select the statement that best represents your thoughts at the beginning of that experience.” The temporal reference of this measure is therefore retrospective. Neither the T1 survey (2023) nor the T2 survey (2025) measured how participants were making meaning of their experience when they completed the questionnaire. Instead, participants in both waves reported what they recalled thinking at the beginning of the stressful experience. Consequently, T1–T2 comparisons concern retrospective reports from two independent samples, and differences may reflect recall, later reinterpretation, or reconstruction of the experience rather than genuine changes in meaning-making processes over time.

#### 2.4.3 PASS

The Athletic Social Support Scale (ASSS; Tsuchiya and Nakagomi, 1994) was used to assess university athletes’ satisfaction with sport-related social support. This 24-item scale consists of four subscales: affective support (nine items), companionship (six items), support for self-esteem (four items), and instructive support (five items).

Examples of affective support questions include “reminding you of what to do now” and “understanding your situation and helping you.” A sample companionship question is “making small talk and having a good time” or “going out and having fun together.” Self-esteem support questions include “recognizing your rivals and building each other up” and “recognizing and appreciating your achievements.” Lastly, instructive support questions include “giving you advice that you can really trust” and “advising you on training plans and other matters to optimize your performance.”

Participants rated these items on a 5-point Likert scale, ranging from 1 (very dissatisfied) to 5 (very satisfied). The total score for each subscale was calculated to represent scores for affective support, companionship, self-esteem support, and instructive support. In the original study, Cronbach’s α was 0.80 for affective support, 0.71 for companionship, 0.52 for support for self-esteem, 0.76 for instructive support, and 0.90 for the total scale. Tsuchiya and Nakagomi (1994) described the factorial validity of the ASSS as relatively high while noting the comparatively low reliability of the support-for-self-esteem subscale. In the present samples, Cronbach’s α values for affective support, companionship, support for self-esteem, and instructive support were 0.95, 0.89, 0.91, and 0.85 at T1, and 0.90, 0.81, 0.82, and 0.79 at T2, respectively (Table 2). Participants completed the ASSS after reading the following instruction: “Based on your interactions with others (people around you) and the support you receive from those you believe are necessary for your sports activities to proceed smoothly, please respond. To what extent are you satisfied with the 24 items listed below? Please select the most appropriate answer from 1 (very dissatisfied) to 5 (very satisfied).” Participants completed the ASSS under the heading “Support from others when experiencing a stressful experience/event related to COVID-19 in athletic club or sport activities.” They were instructed to rate their satisfaction with interpersonal involvement and support from people around them that they considered necessary for smoothly continuing their athletic club or sport activities. Accordingly, PASS was assessed as satisfaction with sport-related support in the context of participants’ COVID-19-related stressful sport experiences. Unlike the meaning-making measure, however, the PASS instruction did not specify a precise temporal point such as the beginning of the experience.

### 2.5 Data analysis

Using multigroup structural equation modeling (MG-SEM), we examined whether the structural associations among meaning-making processes, PASS, and SRG differed between T1 and T2. Because T1 and T2 comprised independent samples and meaning-making was assessed retrospectively with the same reference to the beginning of the stressful experience, these analyses were intended to compare between-wave association patterns rather than within-person psychological change. Data analysis was conducted using SPSS 28.0 and AMOS 30.0. The procedure involved descriptive statistics, reliability and correlation analyses, followed by multigroup comparisons. All tests were two-tailed with α = 0.05. Standardized coefficients (β) are reported for interpretation; z-tests comparing path differences between T1 and T2 were based on unstandardized estimates and their associated standard errors. SRG was the dependent variable, and R^2^ was reported as a measure of explained variance.

Scale and subscale scores were calculated before SEM estimation. Assimilation and accommodation were each represented by their seven-item composite scores and entered as observed variables. PASS and SRG were modeled as latent constructs, with the four ASSS subscale scores (affective support, companionship, support for self-esteem, and instructive support) and the three SRGSA subscale scores (understanding and empathy toward teammates, stress management skills, and attitude change toward sports) serving as indicators, respectively. Reliability was assessed using Cronbach’s α, and Pearson’s product–moment correlations were calculated to describe the relationships among study variables.

#### 2.5.1 Multivariate normality and estimator selection

Prior to structural analyses, multivariate normality of the nine model variables was assessed using Mardia’s (1970) tests of multivariate skewness and kurtosis, implemented in R (*MVN* package, Korkmaz et al., 2014). Results indicated significant violations of multivariate normality at both T1 (Mardia skewness statistic = 467.97, *p* < 0.001; Mardia kurtosis statistic = 15.61, *p* < 0.001) and T2 (Mardia skewness = 737.55, *p* < 0.001; kurtosis = 21.19, *p* < 0.001). Non-normality was pervasive across all subscales, consistent with ceiling effects typical of positively valenced Likert-scaled constructs. Accordingly, the supplementary analyses reported in Sections 3.4 and 3.9 (measurement invariance testing and sensitivity analyses) were conducted using the maximum likelihood estimator with Satorra–Bentler scaling (MLR; Satorra and Bentler, 2001), which provides robust standard errors and a scaled χ^2^ statistic appropriate for non-normal continuous data.

#### 2.5.2 Cross-group coefficient comparisons

For pairwise comparisons of individual path coefficients across T1 and T2 (§3.5), z-tests were based on *unstandardized* parameter estimates using the AMOS Critical Ratios for Differences procedure. Unstandardized coefficients are the appropriate basis for cross-group comparisons, as standardized estimates are rescaled by group-specific variances and are therefore not directly comparable when those variances differ across groups (Millsap, 2011). Standardized estimates (β) are reported throughout the Results section for interpretability.

#### 2.5.3 Sample size adequacy

The present sample sizes (T1: *N* = 145; T2: *N* = 150) are consistent with simulation-based guidelines for structural equation models of comparable complexity. Wolf et al. (2013) demonstrated through Monte Carlo simulation that models with two to three latent factors, three to five indicators per factor, and strong standardized loadings (λ ≥ 0.50) achieve stable parameter recovery and acceptable statistical power at N ≥ 100–150. All factor loadings in the present measurement model substantially exceed this threshold (PASS: λ = 0.80–0.97; SRG: λ = 0.69–0.83), and the model contains two latent constructs with three to four indicators each—placing it within the low-complexity range for which *N* = 145–150 is considered adequate (Kline, 2016). The consistency of key path estimates across multiple model specifications provides additional evidence of parameter stability.

To evaluate model fit, we reported χ^2^ together with the CFI, TLI, RMSEA, GFI, AGFI, and IFI (Abrahim et al., 2019). Because χ^2^ is sensitive to sample size, fit was evaluated using the indices jointly rather than on χ^2^ alone. Values of 0.90 or higher for CFI, TLI, GFI, AGFI, and IFI were treated as acceptable benchmarks (Hair et al., 2014), and RMSEA < 0.10 was used as the prespecified criterion for acceptable fit in the present study (Marsh et al., 2004; Mutumba et al., 2015).

Time point comparisons were conducted through multigroup simultaneous analysis, first confirming whether the same structure held at both time points using a configural invariance model. Subsequently, based on the policy of imposing equality constraints on paths that showed significant differences between T1 and T2, we compared Model 1 (which assumed heterogeneity) with freely estimated paths against Model 2 (which tested homogeneity) with equality constraints placed on “PASS → accommodation” and “accommodation → SRG.” Model differences were primarily assessed using the chi-square difference (Δχ^2^), with ΔCFI and ΔRMSEA referenced as auxiliary measures to determine fit deterioration. Furthermore, regarding between-group differences in each estimate (path coefficient), paths were considered to differ significantly between groups at the 5% level when the value of the test statistic for the difference between parameters was 1.96 or greater in absolute value (Oshio, 2007). It is important to note that the focus of this study was on verifying structural path heterogeneity. As assimilation and accommodation were entered as observed composite variables, formal latent measurement invariance testing was conducted for only the latent constructs PASS and SRG. The results confirmed full scalar invariance for both constructs across T1 and T2 (refer to §3.4), supporting valid comparison of structural path coefficients between time points. Future studies should examine item-level invariance of the meaning-making scale across waves.

## 3 Results

### 3.1 Preliminary analyses: sample comparability and reliability

To examine demographic comparability of the independent T1 and T2 samples, age was compared using an independent-samples Welch t-test, whereas sex, grade level, and sport type were compared using Pearson chi-square tests. Fisher’s exact test with 10,000 Monte Carlo simulations was additionally used for sport type because some expected cell frequencies were <5. Effect sizes were estimated using Cohen’s d for the age comparison and Cramér’s V for the chi-square tests. Years of competitive experience were also compared between waves using an independent-samples t-test. Results for age, sex, grade level, and sport type are presented in Table 1.

Age, sex, and grade level did not differ significantly between T1 and T2 (all *p* ≥ 0.061; effect sizes were small or very small), and mean years of competitive experience were also similar (T1: M = 10.67, SD = 3.47; T2: M = 10.91, SD = 4.32; t(293) = 0.49, *p* = 0.62). Sport type composition, however, differed substantially (χ^2^(26) = 113.79, *p* < 0.001, Cramér’s V = 0.55), with soccer players comprising 57.3% of the T2 sample compared with 14.5% at T1. The samples therefore cannot be regarded as compositionally equivalent, and this difference is addressed in the sensitivity analyses and Discussion.

Internal consistency was generally acceptable at both waves (T1: α = 0.81–0.95; T2: α = 0.79–0.90). Instructive support at T2 (α = 0.79) was marginally below 0.80, and accommodation at T2 (α = 0.80) was at the boundary of that conventional benchmark. The largest decline was observed for accommodation (0.91 at T1 to 0.80 at T2), which should be considered when interpreting between-wave differences involving this variable.

### 3.2 Descriptive statistics and correlations

In the first survey time point (T1: September–October 2023), 298 university students from three universities were surveyed. Of these, 145 athletes (95 (65.5%) males and 50 (34.5%) females; mean age, 19.59 years, SD = 1.06, range, 18–22 years) who met all the inclusion criteria described below were included in the analysis. The grade levels were as follows: 50 first-year students (34.5%), 46 s-year students (31.7%), 42 third-year students (29.0%), and 7 fourth-year students (4.8%). The sport disciplines represented included archery (3, 2.10%), golf (2, 1.40%), soccer (21, 14.50%), tennis (13, 9.00%), basketball (8, 5.50%), badminton (15, 10.30%), volleyball (21, 14.50%), handball (2, 1.40%), boxing (1, 0.70%), bodybuilding (1, 0.70%), ekiden (1, 0.70%), kyudo (1, 0.70%), strength training (1, 0.70%), karate (10, 6.90%), kendo (2, 1.40%), judo (1, 0.70%), swimming (6, 4.10%), baseball (25, 17.20%), and athletics (11, 7.60%). The mean competitive experience was 10.67 years (SD = 3.47).

In the second survey time point (T2: July 2025), independent samples were recruited based on a repeated cross-sectional design. However, one of the universities that participated in T1 was unable to cooperate because of organizational circumstances. Consequently, 236 university students from the remaining two universities were surveyed. Of these, 150 athletes (110 males and 40 females; mean age, 19.36 years, SD = 1.19, range, 18–22 years) who met all the inclusion criteria were included in the analysis. The grade levels were as follows: 58 first-year students (38.7%), 49 s-year students (32.7%), 27 third-year students (18.0%), and 16 fourth-year students (10.7%). The sport disciplines included American football (1, 0.70%), classical ballet (1, 0.70%), soccer (86, 57.30%), softball (7, 4.70%), dance (2, 1.30%), tennis (4, 2.70%), basketball (8, 5.30%), badminton (4, 2.70%), volleyball (7, 4.70%), marching band (1, 0.70%), artistic gymnastics (1, 0.70%), kyudo (2, 1.30%), kendo (4, 2.70%), judo (1, 0.70%), rhythmic gymnastics (1, 0.70%), swimming (3, 2.0%), table tennis (5, 3.30%), baseball (5, 3.30%), and athletics (7, 4.70%). The mean competitive experience was 10.91 years (SD = 4.32).

Table 2 presents the means, standard deviations, reliability coefficients (α), and correlations among the study variables at T1 and T2. PASS subscales were positively correlated with the SRG subscales at both waves (T1 *r* = 0.46–0.62; T2 *r* = 0.44–0.58). Assimilation and accommodation were also positively correlated with the SRG subscales at both waves. Of the nine T1–T2 mean comparisons shown in Table 2, only assimilation differed significantly (T1 M = 25.41, SD = 4.77; T2 M = 23.99, SD = 5.73; *t* = 2.32, *p* < 0.05); the remaining mean comparisons were non-significant.

### 3.3 Model fit at each time point

We first examined the fit of the hypothesized structural model separately at each time point. At T1, χ^2^(23) = 61.261, *p* < 0.001, CFI = 0.966, TLI = 0.947, IFI = 0.967, RMSEA = 0.107, GFI = 0.913, and AGFI = 0.829. The T1 fit was therefore mixed: CFI, TLI, IFI, and GFI met the 0.90 benchmark, whereas AGFI (0.829) was below 0.90 and RMSEA (0.107) exceeded 0.10. We retained the theoretically specified model rather than respecifying it *post hoc*, but the T1 model did not fully meet the fit criteria and the T1 structural estimates should therefore be interpreted with caution, particularly when conclusions depend on T1–T2 differences. At T2, χ^2^(23) = 33.404, *p* = 0.074, CFI = 0.988, TLI = 0.982, IFI = 0.989, RMSEA = 0.055, GFI = 0.953, and AGFI = 0.907, indicating generally good fit.

The path structure at T1 is illustrated in Figure 2.

**Figure 2:**
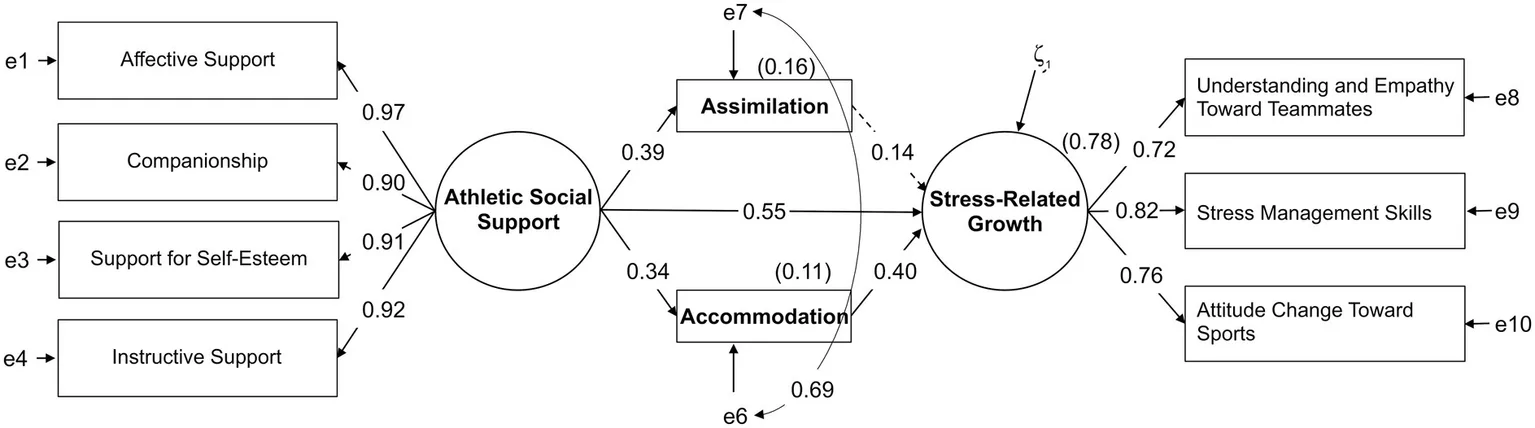
Multigroup analysis of psychological growth following COVID-19-related stress in sport: T1 results. Significant paths are shown by solid lines (*p* < 0.05), whereas dashed arrows denote non-significant paths (n.s.). χ^2^(23) = 61.261, *p* < 0.001, CFI = 0.966, TLI = 0.947, RMSEA = 0.107, GFI = 0.913, AGFI = 0.829, IFI = 0.967. All effects are standardized.

### 3.4 Measurement invariance testing

To evaluate whether the latent PASS and SRG measurement models were comparable across T1 and T2, we conducted stepwise measurement invariance testing (configural → metric → scalar) using lavaan 0.6–19 (Rosseel, 2012) with the MLR estimator. Models were compared using the Satorra–Bentler scaled χ^2^ difference test (Satorra and Bentler, 2001), ΔCFI, and ΔRMSEA. Added equality constraints were considered acceptable when CFI decreased by no more than 0.010 (ΔCFI ≥ − 0.010) and RMSEA increased by no more than 0.015 (ΔRMSEA ≤ + 0.015) (Cheung and Rensvold, 2002).

Results are presented in Table 3. The Satorra–Bentler scaled χ^2^ difference tests were non-significant at both steps (metric vs. configural: Δχ^2^(5) = 2.55, *p* = 0.769; scalar vs. metric: Δχ^2^(5) = 2.92, *p* = 0.712). CFI increased by approximately 0.001 at each step, and RMSEA decreased by 0.010 and 0.009; thus, adding equality constraints did not worsen fit according to the prespecified criteria. These results support scalar invariance of the PASS and SRG measurement models at the subscale-indicator level. Standardized factor loadings in the metric model ranged from λ = 0.69 to 0.97. The invariance analysis therefore supports comparison of structural relations involving the latent PASS and SRG constructs across waves.

**Table 3:** Measurement invariance testing of PASS and SRG across T1 and T2 (configural, metric, and scalar models).

Model	χ^2^	df	*p*	CFI	TLI	RMSEA	90% CI	SRMR	SB Δχ^2^	Δdf	*p*(Δχ^2^)	ΔCFI	ΔRMSEA
Configural	64.13	26	<0.001	0.977	0.963	0.100	[0.069, 0.131]	0.039	—	—	—	—	—
Metric	67.51	31	<0.001	0.978	0.970	0.089	[0.060, 0.118]	0.041	2.55	5	0.769	+0.001	−0.010
Scalar	70.39	36	<0.001	0.979	0.976	0.080	[0.052, 0.108]	0.042	2.92	5	0.712	+0.001	−0.009

Models were estimated using maximum likelihood with robust (Huber–White) standard errors and Satorra–Bentler scaling (MLR). The per-model χ^2^ values are the conventional test statistics returned by lavaan; SB Δχ^2^ denotes the Satorra–Bentler scaled χ^2^ difference test. Added equality constraints were considered acceptable when ΔCFI ≥ − 0.010 and ΔRMSEA ≤ + 0.015 (Cheung and Rensvold, 2002). Analyses used the full T1 + T2 dataset (*N* = 295).

Note on absolute fit: The configural model RMSEA of 0.100 reflects the known tendency of RMSEA to be inflated in models with small degrees of freedom (df = 26) under the MLR estimator, as documented by Kenny et al. (2015) and Shi et al. (2018). The consistent improvement in RMSEA across the invariance hierarchy (from 0.100 to 0.080) and the high CFI/TLI values throughout further support the substantive conclusion of full scalar invariance.

Additionally, the path structure at T2 is illustrated in Figure 3.

**Figure 3:**
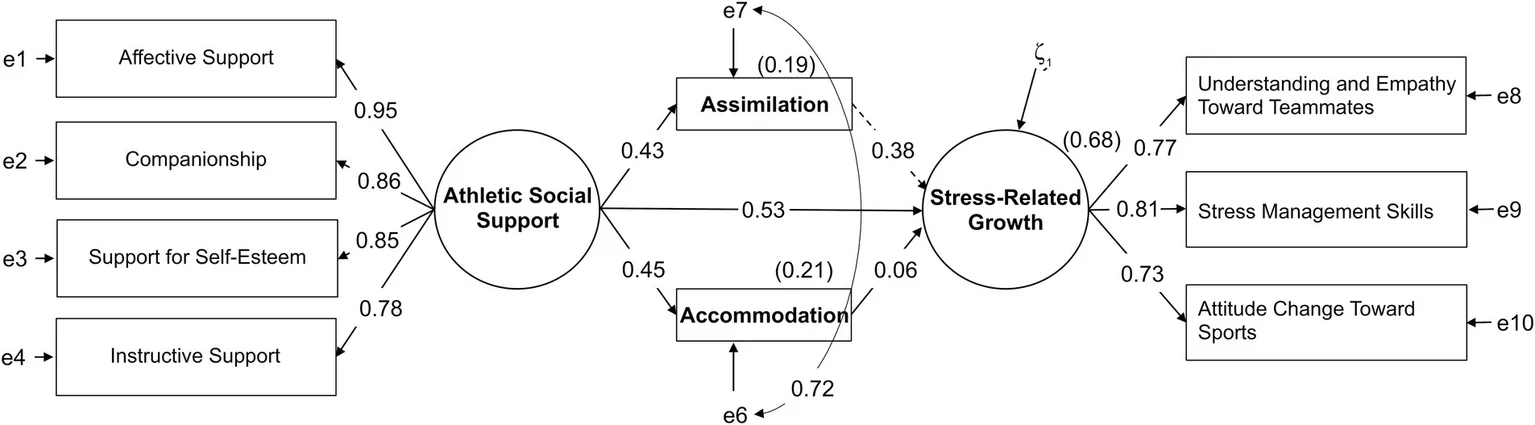
Multigroup analysis of psychological growth following COVID-19-related stress in sport: T2 results. Significant paths are shown by solid lines (*p* < 0.05), whereas dashed arrows denote non-significant paths (n.s.). χ^2^(23) = 33.404, *p* = 0.074, CFI = 0.988, TLI = 0.982, RMSEA = 0.055, GFI = 0.953, AGFI = 0.907, IFI = 0.989. All effects are standardized.

### 3.5 Testing differences between models (test statistics for differences between parameters)

We next compared individual structural paths between T1 and T2 using the critical ratios for differences based on unstandardized estimates (|z| ≥ 1.96 for significance at the 5% level). Two paths differed significantly. The path from PASS to accommodation was larger at T2 (standardized coefficients shown for interpretation: T1 β = 0.34, T2 β = 0.45; z = 2.20), whereas the path from accommodation to SRG was larger at T1 (T1 β = 0.40, T2 β = 0.06; z = −2.83). No other between-wave path difference reached significance. Importantly, although assimilation was not significantly associated with SRG at T1 (β = 0.14) and was significantly associated with SRG at T2 (β = 0.38), the formal between-wave difference in the assimilation-to-SRG path was not statistically significant. Thus, the findings do not establish a statistically significant transition from accommodation to assimilation.

### 3.6 Analysis of equality constraints (relative comparison of models as a whole)

Based on the two paths that differed significantly in the parameter-difference tests, we compared Model 1, in which all paths were freely estimated, with Model 2, in which equality constraints were imposed on the PASS-to-accommodation and accommodation-to-SRG paths. As shown in Table 4, RMSEA and AIC were higher and GFI and CFI were lower in Model 2; Model 1 was therefore retained as the final solution. This comparison supports allowing the identified parameters to vary across waves, but it should not be interpreted as evidence that all paths differ or that the differences represent within-person change over time.

**Table 4:** Results of equality-constraint tests in multigroup SEM.

Model	Model fit indices				
	GFI	AGFI	CFI	RMSEA	AIC
Model 1	0.931	0.879	0.977	0.055	174.706
Model 2	0.925	0.872	0.972	0.060	183.102

### 3.7 Time point-specific paths in the final model (Model 1)

We report the final freely estimated model (Model 1) separately for T1 and T2. Standardized factor loadings were high for SRG (T1: 0.72–0.82; T2: 0.73–0.81) and PASS (T1: 0.90–0.97; T2: 0.78–0.95). The coefficients of determination for SRG were R^2^ = 0.78 at T1 and R^2^ = 0.68 at T2. Correlations among the observed study variables are reported in Table 2 and are not repeated here.

In the structural part, at T1, the paths from PASS to assimilation (β = 0.39) and from PASS to accommodation (β = 0.34) were significant. The path from assimilation to SRG was not significant (β = 0.14), whereas the path from accommodation to SRG was significant and positive (β = 0.40), and the direct path from PASS to SRG was also large and significant (β = 0.55). At T2, the paths from both PASS to assimilation (β = 0.43) and PASS to accommodation (β = 0.45) were significant; the path from assimilation to SRG was significant and relatively large (β = 0.38), whereas the path from accommodation to SRG was not significant (β = 0.06). The direct path from PASS to SRG remained large and significant (β = 0.53).

In summary, the within-wave pattern differed: accommodation was significantly associated with SRG at T1 but not at T2, whereas assimilation was significantly associated with SRG at T2 but not at T1. However, formal between-wave testing identified a significant difference for the accommodation-to-SRG path but not for the assimilation-to-SRG path. Hypothesis 1 therefore received partial rather than full support. PASS showed a large direct association with SRG at both waves, consistent with the direct-association component of Hypothesis 2. These findings are best described as between-sample differences in association patterns rather than as a temporal shift from accommodation to assimilation.

#### 3.7.1 Effect size interpretation

For descriptive purposes, the direct PASS-to-SRG path was large at both time points (T1: β = 0.55; T2: β = 0.53). The accommodation-to-SRG coefficient was 0.40 at T1 and 0.06 at T2, consistent with the significant between-wave test for this path. The assimilation-to-SRG coefficient was 0.14 at T1 and 0.38 at T2, but this descriptive difference was not statistically significant in the formal between-wave comparison. R^2^ for SRG was 0.78 at T1 and 0.68 at T2. These values characterize the two models; they should not be interpreted as evidence of developmental progression, and interpretation of the T1 estimates should remain cautious because of the mixed T1 model fit.

### 3.8 Supplementary bootstrap indirect-effect analysis

The supplementary bootstrap analyses were retained only to summarize model-implied indirect associations within each wave. They were conducted separately for T1 (*N* = 145) and T2 (*N* = 150) using lavaan 0.6–19 with B = 2,000 bootstrap resamples and bias-corrected (BC) 95% confidence intervals obtained with lavaan’s bca.simple option, which applies bias correction without an acceleration correction. Because PASS, meaning-making, and SRG were assessed within the same cross-sectional survey and were not temporally ordered within participants, these quantities are products of associations specified in the model and are not interpreted as evidence that social support leads to SRG through meaning-making.

The supplementary models showed fit below conventional thresholds in both groups (T1: CFI = 0.887, RMSEA = 0.193; T2: CFI = 0.869, RMSEA = 0.181). Accordingly, these results are treated as supplementary and interpreted cautiously; the primary basis for inference remains the structural path estimates and formal between-wave comparisons reported above.

Indirect estimates and BC 95% confidence intervals are reported in Table 5.

**Table 5:** Bootstrap-based indirect effects of PASS on SRG via assimilation and accommodation at T1 and T2.

Time point	Path	Unstd. indirect β	SE	95% BC CI	Significant?
T1 (*N* = 145)	PASS → Assimilation → SRG	0.051	0.040	[−0.018, 0.139]	No
T1 (*N* = 145)	PASS → Accommodation → SRG	0.127	0.045	[0.057, 0.241]	Yes
T1 (*N* = 145)	Total indirect effect	0.178	0.048	[0.089, 0.282]	Yes
T2 (*N* = 150)	PASS → Assimilation → SRG	0.223	0.075	[0.109, 0.403]	Yes
T2 (*N* = 150)	PASS → Accommodation → SRG	0.032	0.076	[−0.098, 0.208]	No
T2 (*N* = 150)	Total indirect effect	0.254	0.091	[0.113, 0.487]	Yes

Supplementary bootstrap indirect effects (BC 95% CIs). Indirect effects are unstandardized (product of unstandardized a and b paths). Estimator = ML; se = “bootstrap”; B = 2,000. Direct path PASS → SRG: T1 β(std) = 0.576, *p* < 0.001; T2 β(std) = 0.553, *p* < 0.001. PASS, Perceived Available Support in Sport; SRG, Stress-Related Growth.

At T1, the model-implied indirect association of PASS with SRG through accommodation was 0.127 (BC 95% CI [0.057, 0.241]), whereas that through assimilation was 0.051 (BC 95% CI [−0.018, 0.139]). At T2, the corresponding estimates were 0.032 through accommodation (BC 95% CI [−0.098, 0.208]) and 0.223 through assimilation (BC 95% CI [0.109, 0.403]). These within-wave patterns do not constitute evidence of causal mediation, and the separate analyses do not test whether an indirect association itself differed significantly between T1 and T2.

### 3.9 Sensitivity analyses: sport composition

Given the significant difference in sport type composition between T1 and T2—with soccer players comprising 57.3% of the T2 sample (*n* = 86) versus 14.5% at T1 (*n* = 21)—we conducted sensitivity analyses to evaluate whether the T2 structural pattern is robust to this composition imbalance. Three models were estimated using the MLR estimator:

- Model A (reference): T2 full sample (*N* = 150), replicating the primary T2 analysis.
- Model B: T2 excluding soccer players (*N* = 64 non-soccer participants).
- Model C: T2 full sample (*N* = 150) with soccer versus other sports as a binary covariate (0 = other, 1 = soccer) entered on assimilation, accommodation, and SRG.

Results are presented in Table 6. The principal T2 pattern was similar across the three specifications. The indirect association of PASS with SRG via assimilation remained significant across Models A, B, and C (β = 0.172, 0.182, and 0.156, respectively; all *p* ≤ 0.007), whereas the indirect association via accommodation remained non-significant (β = 0.024, 0.088, and 0.036; all *p* ≥ 0.27). The direct association of PASS with SRG was also similar across models (β = 0.51–0.55). These sensitivity analyses reduce, but do not eliminate, concern that the T2 findings may be influenced by the overrepresentation of soccer players, particularly because the non-soccer subgroup was small and the samples remained compositionally different across waves.

**Table 6:** Sensitivity analyses of structural pathways across sport composition models (T2).

Path	Model A (T2 full) β(std) *p*	Model B (T2 excl. soccer) β(std) *p*	Model C (T2 + sport cov.) β(std) *p*
PASS → Assim (a1)	0.458 < 0.001	0.511 < 0.001	0.447 < 0.001
PASS → Accom (a2)	0.471 < 0.001	0.521 < 0.001	0.471 < 0.001
Assim → SRG (b1)	0.376 < 0.001	0.357 0.004	0.348 < 0.001
Accom → SRG (b2)	0.052 0.652	0.169 0.268	0.077 0.485
PASS → SRG (direct)	0.553 < 0.001	0.512 < 0.001	0.542 < 0.001
Indirect via Assim	0.172 < 0.001	0.182 0.007	0.156 0.001
Indirect via Accom	0.024 0.654	0.088 0.272	0.036 0.488
Soccer → SRG	—	—	0.185 0.006

Sensitivity analysis: standardized path coefficients across T2 model specifications. Model B: *N* = 64 (small-sample estimates should be interpreted with caution). Indirect effects estimated using the delta method (MLR estimator). Assim, assimilation; Accom, accommodation; PASS, perceived available support in sport; SRG, stress-related growth.

One finding in Model C warrants specific note: when soccer participation is included as a covariate, it shows a significant positive direct association with SRG (β = 0.185, *p* = 0.006), independent of PASS and the two meaning-making pathways. The present data do not identify the mechanism underlying this association; any explanation for the higher SRG among soccer players remains speculative. This finding reinforces the importance of acknowledging the sport-composition imbalance as a boundary condition on the generalizability of T2 findings.

## 4 Discussion

### 4.1 Main findings of the present study

This repeated cross-sectional study compared associations among PASS, assimilation, accommodation, and SRG in two independent samples of Japanese university athletes surveyed in 2023 and 2025. Three findings are central. First, accommodation was significantly associated with SRG at T1 but not at T2, whereas assimilation was significantly associated with SRG at T2 but not at T1. Formal between-wave testing, however, identified a significant difference for the accommodation-to-SRG path but not for the assimilation-to-SRG path; Hypothesis 1 was therefore only partially supported. Second, PASS showed a large and similar direct association with SRG at both waves. Third, the supplementary indirect estimates followed the within-wave pattern but do not establish causal mediation. Overall, the results describe heterogeneity in association patterns across two independent samples; they do not demonstrate that individual athletes progressed from accommodation to assimilation over time.

### 4.2 Between-wave differences in associations involving meaning-making

The T1 association between accommodation and SRG is compatible with accounts in which stressful experiences are accompanied by revision of beliefs and goals, whereas the T2 association between assimilation and SRG is compatible with accounts in which experiences are integrated into existing frameworks (Joseph and Linley, 2005; Payne et al., 2007; Park, 2010). These interpretations are theoretically plausible, but the present design cannot establish that one process replaced the other over time.

The most important alternative explanation is retrospective reconstruction. Participants in both 2023 and 2025 were asked to report what they recalled thinking at the beginning of their COVID-19-related stressful experience, rather than their current meaning-making at the survey date. Differences between waves may therefore reflect differences in recall, later reinterpretation, or reconstruction of past experiences. In addition, the samples were independent and differed in sport composition; one of the three T1 universities did not participate at T2; unmeasured cohort and contextual differences may have contributed; and the mixed T1 model fit adds uncertainty to contrasts that depend on the T1 estimates. These factors prevent the observed heterogeneity from being attributed uniquely to developmental change.

Howard-Cook and Howells (2024) further illustrate why reports of growth after COVID-19 should be interpreted cautiously: their qualitative study of elite athletes distinguished potentially real and illusory adversarial growth and emphasized athlete well-being. Accordingly, SRG scores in the present study are treated as meaningful self-perceptions of positive change rather than as objectively verified transformation.

### 4.3 Role of PASS

The direct association between PASS and SRG was large at both time points (T1: β = 0.55; T2: β = 0.53), making this the most consistent finding of the study. This pattern is consistent with prior evidence linking social support with PTG/SRG across contexts (Helgeson et al., 2006; Prati and Pietrantoni, 2009; Galli and Reel, 2012; Nakamura and Tsuchiya, 2020). However, the present data do not establish that support causes SRG. The significant between-wave difference in the PASS-to-accommodation path likewise cannot be interpreted as a change in how support operated within the same athletes because the samples were independent.

### 4.4 Positioning in the COVID-19 context

The present findings should be understood in the context of prolonged adjustment following COVID-19-related sport disruption rather than as evidence of an initial-versus-later developmental sequence. Differences in training continuity, competition opportunities, social contact, role stability, and other contextual conditions between the periods surrounding T1 and T2 may have contributed to the observed associations, but these factors were not measured directly.

### 4.5 Theoretical implications

The results are consistent with the view that SRG is associated with multiple cognitive and interpersonal factors rather than a single fixed process (Tedeschi and Calhoun, 2004; Park, 2010). At the same time, structural differences between independent samples identify where associations differ, not why they differ. Longitudinal data assessing current meaning-making in the same athletes are required before claims about within-person developmental change or temporal sequencing can be made.

### 4.6 Practical implications

The most defensible practical implication is the consistent positive association between perceived sport-related support and SRG in both samples. Coaches, athletic staff, and sport psychology practitioners may therefore attend to supportive relationships when helping athletes respond to prolonged stress. However, the present data do not show that increasing PASS causes SRG and do not justify recommending accommodation-focused strategies at one phase and assimilation-focused strategies at another. Such phase-specific approaches should be treated as hypotheses for future longitudinal and intervention research.

### 4.7 Methodological considerations and limitations

Several limitations are particularly important for interpreting the present results. First, the T1 model fit was mixed: although CFI, TLI, IFI, and GFI were acceptable, AGFI (0.829) was below 0.90 and RMSEA (0.107) exceeded 0.10. This reduces confidence in the T1 structural estimates and warrants caution in conclusions based on T1–T2 differences. Second, the supplementary indirect-effect models fit poorly, and the variables were measured cross-sectionally within each wave, so the indirect estimates cannot demonstrate causal mediation. Third, meaning-making was retrospective at both waves, making recall, reinterpretation, and reconstruction viable explanations for between-wave differences. Fourth, the two samples were independent and differed substantially in sport composition (soccer: 57.3% at T2 vs. 14.5% at T1; Cramér’s V = 0.55); although sensitivity analyses produced a similar principal T2 pattern, they do not eliminate this comparability concern. Fifth, one T1 university did not participate at T2, and no individual-level institution identifier was retained, so institutional differences could not be examined directly. Finally, all variables were self-reported, and the study was conducted within Japanese collegiate sport, which should be considered when generalizing the findings.

### 4.8 Future directions

Future studies should follow the same athletes longitudinally and assess current meaning-making, PASS, and SRG at each wave. Such designs would allow within-person change and temporal ordering to be examined separately from retrospective reconstruction. Future work should also recruit samples that are more comparable across sports and institutions and should test any phase-specific meaning-making or support intervention only after prospective evidence identifies plausible temporal mechanisms.

## 5 Conclusion

Among university athletes reporting COVID-19-related sport stress, PASS and meaning-making showed different patterns of association with SRG across two independent samples. Accommodation was associated with SRG at T1 and assimilation at T2, but the formal between-wave difference was significant for the accommodation-to-SRG path and not for the assimilation-to-SRG path. PASS showed a large positive association with SRG at both waves. Given the repeated cross-sectional design, retrospective meaning-making assessment, mixed T1 model fit, and sample-composition differences, the findings do not demonstrate a within-person shift from accommodation to assimilation or a causal mediation process. They instead identify between-sample heterogeneity in meaning-making associations and a stable association between perceived sport-related support and SRG that should be examined prospectively.

## Data Availability

The raw data supporting the conclusions of this article will be made available by the authors, without undue reservation.
